# Use of the Sphenopalatine Ganglion Block to Treat Migraine Headaches in the Emergency Department

**DOI:** 10.7759/cureus.21428

**Published:** 2022-01-19

**Authors:** Aaron Morgan, Gennaro Romanello

**Affiliations:** 1 Emergency Medicine, University Hospitals Community Consortium, St. John Medical Center, Westlake, USA

**Keywords:** common emergency department complaints, nerve block, migraine treatment, sphenopalantine ganglion block, migraine, headache disorders

## Abstract

Headaches are a common presenting complaint to the emergency department. Amongst the common non-life-threatening headaches, migraine headaches tend to be one of the more severe. Commonly, migraines are treated with the so-called “migraine cocktail.” At our facility, this cocktail is usually IV fluids, metoclopramide, diphenhydramine, and ketorolac. The patient is then left in a quiet, dark room for two to three hours, then reassessed for symptom improvement or if more medications are required. Recently we have begun to employ the sphenopalatine ganglion (SPG) block as a rapid, and easy-to-administer alternative to the classic migraine cocktail. The SPG block can often provide sufficient improvement of symptoms within 15 minutes to allow the patient to be discharged. Patients are grateful to have such rapid pain relief, and the ED flow is improved with a door-to-discharge time that can be less than an hour. The following is a series of three recent cases where we have used the SPG block to treat migraines in our ED patients.

## Introduction

The Headache Classification Committee of the International Headache Society recognizes three broad categories of headaches, namely primary headaches, secondary headaches, and finally a third catchall category called “painful cranial neuropathies, other facial pain, and other headaches.” Migraines fall into the primary headache category. Migraines can be with or without an aura, and episodic or chronic in nature [1]. There are many treatment modalities being used for migraines, including non-steroidal anti-inflammatory drugs (NSAIDs), opioids, dopamine receptor antagonists (metoclopramide, prochlorperazine, etc.), ketamine, propofol, ergots, triptans, nerve blocks, non-pharmacologic treatments (nutraceuticals, acupuncture, transcutaneous cranial nerve stimulation, etc.), and the list goes on [2-6]. Everyone seems to have their own theory on the best way to treat migraines. Some of these options have good data supporting their use, such as dopamine receptor agonists and NSAIDs [7-9], whereas other treatments currently lack high-quality data, as is the case with daith piercing [10].

The SPG block was first described in the early 1900s by Dr. Sluder. Originally, he used a cocaine-soaked pledget on the end of a wire to anesthetize the ganglia in patients with a constellation of symptoms including neurologic, motor, sensory, and gustatory symptoms he referred to as “Sluder’s Neuralgia” [11]. The SPG block is currently used to treat a variety of painful conditions of the head including cluster headaches, trigeminal neuralgia, postdural puncture headaches, and importantly for our purposes, migraines [12]. Since the early 1900s, several different methods have been used to perform the SPG block. Some require complex equipment such as fluoroscopy, or specialized catheters. Others are extremely simple and do not vary much from Sluder’s original procedure [13].

There are no large-scale prospective studies in the literature examining the use of the SPG block for migraines in the ED. The closest to this would be a study conducted in 2015 that evaluated the use of a specialized aerosolization device called the Tx360 nasal applicator (Tian Medical LLC, Libertyville, IL) to perform the SPG block on patients in the ED with what the authors referred to as “acute anterior headaches” [14]. They ultimately enrolled 87 patients with 41 in the treatment group and 46 in the control group. In their study, just under half of the patients in the treatment group had greater than a 50% reduction in their pain scale at 15 minutes, which was not significantly more than the control group [14]. However, it is unclear if this represented a failure of the SPG block as a treatment modality, the delivery device itself, or the choice of anesthetic which was 0.3 mL of 0.5% bupivacaine. In another study conducted in a neurology clinic that specifically targeted migraines had better success rates. Their retrospective review of 55 patients found 70.9%, 78.2%, and 70.4% were “headache-free” at 15 minutes, two hours, and 24 hours respectively [15]. In that study, they used a specialized catheter called the Sphenocath (Dolor Technologies LLC, Clearfield, UT) to deliver 2mL of 2% lidocaine, in a very similar manner to the technique that we employed, though we did not use any specialized equipment as detailed below.

The first several times I performed the procedure, I used a long cotton-tipped applicator readily available in any ED. The cotton-tipped applicator would be soaked in a topical anesthetic, such as 4% viscous lidocaine, and inserted through the nares, above the middle turbinate until it reached the area of the ganglion. This procedure worked adequately but was often not tolerated well by the patients. After reviewing the literature, and speaking with local pain management specialists, we started using the following procedure.

Adult patients with a normal neurologic exam, without focal neuro deficits, who are at their cognitive baseline, can understand the procedure, and if there was no concern for a life-threatening headache in the opinion of the treating provider, are considered for the SPG block. Absolute contraindications include allergic reaction to the medications and nasal/sinus surgery or trauma within the previous six weeks. Relative contraindications include the use of anticoagulants and frequent or habitual intranasal cocaine use due to the increased risk of epistaxis. Once the above criteria are confirmed, verbal consent is obtained during which we describe the procedure and common side effects. The patient is warned about the bitter taste of the anesthetic and cautioned that the back of the throat will be numb for about two hours when 2% lidocaine is used. A small number of patients will experience lightheadedness. There are certain physiologic responses that will be evident when the lidocaine is in the right location. You will see lacrimation, conjunctival injection, and a flushing of the cheeks. When this block is performed in pain management or neurology clinics, they will often place skin temperature probes on the bilateral zygoma. It is expected to see an increase in the skin temperature in the V2 distribution of the trigeminal nerve of 3-5 degrees Fahrenheit. It is important to warn the patient of these responses before you begin. The patients are always given the choice upfront between the SPG block and the migraine cocktail. If they elect to go with the SPG block, the following procedure is followed.

The patient is laid down supine in slight Trendelenburg, with a shoulder roll to achieve the proper sniffing positioning of the head. The head is then turned 15-20 degrees to the side being treated. A 1.8 inch long 20-gauge angiocatheter sheath (without the metal introducer needle) is inserted in the nares following the angle of the bridge of the nose. If the nasal bone is gently contacted, the angiocatheter is pulled back a millimeter or two, and 2mL of 2% lidocaine is instilled into the area. If properly positioned, the lidocaine will flow over the middle turbinate and collect in the pterygopalatine fossa that holds the ganglion. The patient is then left in that position for one to two minutes, after which the procedure is repeated on the opposite side. The patient is left in the Trendelenburg position for about 10-15 minutes and their pain reassessed. If the patient has little to no response from the SPG block, they are then offered a migraine cocktail. If they have partial but incomplete/insufficient relief, the patient is typically offered their choice of a repeat procedure or alternatively, the migraine cocktail.

The above procedure was taught to the emergency medicine residents and attendings at the St. John Medical Center in Westlake, Ohio as part of an ongoing quality improvement project. What follows is a series of three cases from our experience with the SPG block used to treat migraines in the emergency department.

## Case presentation

Case 1

A 34-year-old female came into the ED complaining of a migraine consistent with her typical headaches. Other than her recurrent, intermittent migraines, she denied chronic medical problems. She was seen and treated in the ED about two weeks prior to this encounter for the same complaint and her migraine resolved after IV fluids, metoclopramide, diphenhydramine, and oxycodone/acetaminophen. After the first visit, the patient followed up with a neurologist and received a prescription for Fioricet. She had tried the Fioricet prior to this visit with no relief so she came into the ED for further treatments. She described her headache as bilateral, parietal, and throbbing in nature. She reported the pain as 8/10 and denied any other associated symptoms. She was afebrile and her vital signs were within normal limits. Cranial nerves II-XII were tested, along with a complete neuro physical examination. The patient had no focal neurologic findings and no indication for neuroimaging. Her presentation and physical exam were reassuring without findings to suggest a life-threatening intracranial problem.

The SPG block was described to the patient, and she was agreeable to trying it. Immediately after instillation of the lidocaine, the patient reported that her pain improved to a 6/10. The patient was reassessed after 15 minutes, and her migraine had completely resolved. She was discharged from the ED shortly afterward.

Case 2

A 33-year-old female with a history of migraines and polycystic ovarian syndrome presented to the ED complaining of a migraine and vomiting. She reported that her current migraine episode was typical for her in its location, quality, and associated nausea and vomiting. She had previously been on rizatriptan for her migraines which worked well but had lost her insurance and had not been able to refill her prescription for quite a while. Recently she had been taking over-the-counter Excedrin for her migraines which often helped, though her current migraine was persistent despite the treatment. Other than nausea and vomiting, she had no other associated symptoms. The patient had a complete physical and neurological examination. There were no abnormalities identified, including no focal neurologic findings. The patient’s presentation and exam were reassuring for a non-life-threatening headache. There was no indication for neuroimaging.

The patient was given ondansetron and her vomiting improved. The SPG block was described to her, and she agreed to try it. The SPG block was performed using the procedure described above. Within minutes, the patient’s pain improved from an 8/10 to a 2/10. She was satisfied with the improvement in her symptoms. She was discharged home with a new prescription for rizatriptan and a referral to a new neurologist.

Case 3

This next case is a little different than the earlier ones but highlights the versatility of this treatment. A 23-year-old female with a history of asthma presented to the ED with a persistent headache for the past month. Two months before this visit, the patient was involved in a car crash during which she had struck her head on the window and suffered a brief loss of consciousness. She first sought care for these headaches three days after the accident. She underwent a full exam including CT scans of her head and neck. The imaging was completely within normal limits, with no acute findings. Her physical exam only showed muscle spasms in her neck. The patient had been discharged with prescriptions for lidocaine patches, cyclobenzaprine, and naproxen, as well as a neurology follow-up. She was advised to return for new or worsening symptoms. During the first month after the crash, the patient had similar intermittent headaches that typically resolved on their own. However, her current headache had been constant for about a month by the time she presented to the ED. The headache was global, 8/10, associated with nausea and “brain fog”. The symptoms had been waxing and waning over the previous month but were always present to one degree or another. She had a follow-up appointment with neurology, but it was still a few weeks away. A thorough neurologic exam was performed, including testing of cranial nerves II-XII, motor function testing, and sensation testing in all extremities. No focal neurologic findings were present. Despite a benign neuro exam and a previously normal head CT on her first visit just after the crash, the patient was offered a repeat head CT due to the prolonged nature of the headache. She chose to decline the repeat imaging. Overall, the patient’s history, presentation, and examination were not suggestive of a life-threatening headache, and the provider was comfortable with forgoing the neuroimaging on this visit.

Once again, treatment options were presented, and the patient elected to try the SPG block. The block was performed in our usual fashion, and the patient was pain-free within 10 minutes of the administration of the procedure. It was suspected that the patient’s migraine was likely the result of post-concussive syndrome, which was later confirmed by neurology during her outpatient follow-up. The patient was very relieved to be pain-free for the first time in a month.

## Discussion

According to the Global Burden of Disease studies, migraines are the third most common disease in the world [16]. About 15% of American’s overall, and just over one in five women in the U.S. report suffering from migraines [16]. In 2016 alone, this led to about 4 million ED visits for headaches, making this the fifth most common reason for a visit to the ED [16]. Due to the commonality of migraines, the economic burden begins to add up very quickly. Between direct healthcare costs and the cost of lost wages and productivity, it is estimated that the total economic impact of migraines in the U.S. is between $13 and $17 billion per year [17].

We clearly need additional treatment options that are effective, inexpensive, and with few or mild side effects. I believe that the SPG block should be one of the options regularly employed in this role. It is not 100% effective, nor is it without potential side effects. Though that can be said of any other treatment as well. However, compared to the traditional migraine cocktail, the SPG block is less expensive, faster, and has a good side effect profile. In our experience, side effects have been mild including the above-mentioned bitter taste, lacrimation, and numbness in the back of the throat which is similar to other published reports [18]. We have also had one patient report lightheadedness after the SPG block was performed, though they still felt well enough to go home shortly after the block.

Once performed, the provider and patient will know within a few minutes if the SPG block is going to be effective. Most patients will begin to feel relief within two to three minutes, and the full effect of the treatment is typically seen in no more than 15 minutes. If it fails, the provider simply orders their preferred migraine cocktail, and the only thing lost is a little bit of time, and minimal expense (our pharmacy reported the cost of a 5mL vial of 2% lidocaine at $1.18). This article highlights using the SPG block for migraines, though I have also successfully used it to treat trigeminal neuralgia, the pain from sinusitis, and once I treated the pain from a spontaneous subarachnoid hemorrhage with it. The SPG block that we utilized can be taught in half an hour and uses equipment already in common use in every ED in the country.

The SPG block has become a favorite of many of the residents and attendings at our institution. I asked my fellow residents and attendings to provide me with some data on the success rates of the SPG block for their patients based on a review of their procedure logs over the preceding six months. This gave us a sample of 16 patients, age 19 to 65 (average age 36), who had before and after pain scores recorded. Of these patients, 12 had at least a 50% reduction in the post-procedure pain score. This gives us a 75% success rate for our small sample size which is similar to previous studies [15]. It was also noted that most patients seem to have an “all or nothing” response. For those patients who did have relief from the block, the pain reduction tended to be nearly complete, with 8 of the 12 reporting 100% pain relief. Most of the unsuccessful blocks usually have little to no improvement at all. Given the all-or-nothing response, future research on identifying the specific characteristics that make the SPG block more likely to be successful would be worthwhile. Unfortunately, the data for a number of patients I received from my colleagues did not include both before and after pain scores, precluding their use in the above totals. This is an obvious limitation of this type of review. Also, due to limitations in our electronic medical records (EMR)/ software, it was not possible to run a query for all patients that received the SPG block since we started to implement it in our program. Therefore, we chose to present the information as a short case series instead of a more rigorous retrospective review. Our program is planning to perform formal prospective studies to better quantify these admittedly anecdotal findings in the future. Additional research should also consider examining the degree to which the SPG block can impact the overall ED flow (by improved room turnover times), patient satisfaction, and a cost analysis compared to more traditional approaches should be looked at. A cost analysis would need to include not only the cost of the medications, IV tubing, and other equipment but also the manpower cost of having a nurse available to periodically check on the patient during the hours of waiting for the migraine cocktail to work.

## Conclusions

The SPG block is a useful tool that every ED physician should consider adding to their toolbox for migraine treatment and potentially other sources of cephalgia as well. There remain many questions that still need to be examined. However, I believe there is enough evidence for the 100+ year old SPG block to be routinely used in the ED. I am not suggesting that the SPG block will replace all other treatments. Nor do I think it should be a “last ditch effort” if nothing else is working. Instead, I would urge the reader to consider employing the SPG block early in the treatment of migraines, or even as a first line option offered to the patient with shared decision making. The SPG block is simple, quick, often effective, and deserving of your consideration for your next migraine patient.
